# Human capital sustainability leadership: From personality traits to positive relational management

**DOI:** 10.3389/fpsyg.2023.1110974

**Published:** 2023-01-19

**Authors:** Annamaria Di Fabio, Antonia Bonfiglio, Letizia Palazzeschi, Alessio Gori, Andrea Svicher

**Affiliations:** ^1^Department of Education, Languages, Intercultures, Literatures and Psychology (Psychology Section), University of Florence, Florence, Italy; ^2^Department of Health Sciences (Psychology Section), University of Florence, Florence, Italy

**Keywords:** human capital sustainability leadership, positive relational management, personality traits, healthy organizations, healthy workers, healthy business, sustainable development, strength-based prevention perspectives

## Abstract

**Introduction:**

Constructing positive and supportive relationships is fundamental for healthy workers and healthy organizations and to cope with the current challenging work scenario. Organizations need to acknowledge the relevance of the relationships for workers and organizational well-being and adopt managing practices that enhance healthy relationships for sustainability and sustainable development.

**Methods:**

The current research sought to investigate the associations between positive relational management (PRM) and human capital sustainability leadership (HCSL), taking into account personality traits. The big five questionnaire (BFQ), the PRM Scale (PRMS), and the HCSL Scale (HCSLS) were administered to 191 Italian workers.

**Results:**

Findings displayed that PRM was able to add incremental variance over personality traits referring to HCSL.

**Discussion:**

In terms of strength-based prevention perspectives for healthy organizations, PRM may be a favorable construct linked to HCSL.

## Introduction

The current era is marked by a challenging work landscape with incessant ongoing transitions (Peiró et al., 2014), endangering the workers’ well-being (Di Fabio and Kenny, 2016b; Blustein et al., 2019). Constructing positive and supportive relationships is fundamental for healthy workers, healthy business, and healthy organizations (Di Fabio et al., 2020) in the current challenging scenario. Organizational environments need to recognize the value of the relationships for workers and organizational well-being (Blustein, 2011), as well as for reducing dysfunctional work-related correlates, such as occupational fatigue (Di Fabio et al., 2021), perfectionism (Di Fabio et al., 2022) fostering relevant assets (Di Fabio and Palazzeschi, 2012; Di Fabio et al., 2017; Palazzeschi et al., 2018; Duradoni and Di Fabio, 2019; Gori et al., 2022; Svicher et al., 2022a,b). It asks for incorporating managing practices that enhance healthy relationships for sustainability and sustainable development (Di Fabio and Peiró, 2018). In this framework the value of innovative leadership styles for Sustainability Science is recognized (Di Fabio, 2017a). The human capital sustainability leadership (HCSL; Di Fabio and Peiró, 2018) is an actual concept developed in the field of the psychology of sustainability and sustainable development (Di Fabio, 2017b; Di Fabio and Rosen, 2018) that introduces the psychological perspective as a lens to better understand processes related to the sustainable developmental goals issues (Di Fabio and Rosen, 2018), contributing to the trans-disciplinarity of the sustainability science (Rosen, 2009; Dincer and Rosen, 2013). This style of leadership is based on a higher order model (including sustainable, ethical, mindful, servant leadership) that aims at promoting flourishing and resilient workers and at enhancing healthy organizations implementing a positive circuit of performance and long-term well-being (Di Fabio and Peiró, 2018).

In the psychology of sustainability and sustainable development framework it is recognized the importance of the strength-based prevention approach (Di Fabio and Saklofske, 2021), also considering primary prevention actions (Hage et al., 2007; Di Fabio and Kenny, 2015, 2016a), with a focus on enhancing positive resources increasable through trainings, unlike traits of personality usually considered more fixed (Costa and McCrae, 1992). Centering on positive resources for workers (Di Fabio et al., 2020) turned out to be essential for promoting healthy organizations. In this framework, managing positive relationships at work could be crucial for healthy and sustainable organizations (Di Fabio, 2017a, 2017b).

Relationships and relational experiences at work emerged as crucial aspects for successful performances (Kenny et al., 2003; Blustein, 2006). With reference to the relational theory of working (Blustein, 2011) and the Psychology of Working Framework and Theory (Blustein, 2013; Duffy et al., 2016), work is considered as an inherently relational act. The relational interactions among workers might therefore be regarded as essential aspects, emphasizing the relevance of generating positive circumstances to sustain optimal social connections in organizational settings in terms of flourishing relationships (Blustein et al., 1995; Blustein, 2013; Di Fabio, 2016). The goal should therefore be to facilitate individuals in building lives *via* work and relationships (Di Fabio and Blustein, 2016) with a positive-oriented approach which emphasizes the value of constructing positive relationships and support in the work environments (Di Fabio, 2017a). Positive relationships are a fundamental factor for the well-being of individuals (Blustein, 2011) and promoting positive relationships can increase well-being at work also fostering characteristics of decent work (Svicher et al., 2022a).

The relevance of a condition of well-being of human resources at work includes improving workers’ opportunities to be able to adapt themselves to the shifting conditions and fluidity of the contemporary scenario (Di Fabio and Gori, 2016a, 2016b; Di Fabio and Peiró, 2018). In this current framework the value of positive relational management (PRM) emerged (Di Fabio, 2016). The construct of PRM (Di Fabio, 2016) is a strength for relational positive and productive adaptation to the context. It is constituted of three factors namely Respect, Caring, and Connectedness (Di Fabio, 2016). Each factor is evaluated considering the balance between the individual and others including reciprocity in relationships (Di Fabio, 2016). Thus, PRM goes beyond the traditional idea of social support, conceiving respect, caring, and connectedness as arising from the balance in and the reciprocity of three specific areas of self-perceptions: (1) The individual for other people; (2) the other people for the individual; (3) the individual for him/herself (Di Fabio, 2016; Di Fabio and Saklofske, 2019). In this way, PRM is configured as a promising resource in strength-based prevention perspectives (Di Fabio and Saklofske, 2021) as well as in a primary prevention approach (Di Fabio and Kenny, 2015).

HCSL (Di Fabio and Peiró, 2018) is a novel higher-order construct. It goes beyond the classic concept of sustainable leadership by placing itself in an integrated positive direction. HCSL incorporates other contemporary aspects of leadership relevant to the functioning and growth of human capital, in line with a psychological and sustainable point of view (Di Fabio and Peiró, 2018). This current construct emphasizes healthy workers and healthy organizations as characterized by success and sustained by the positive circuit of long-term well-being and performance. Its higher-order structure integrates the new concept of sustainability leadership with ethical leadership, mindful leadership, and servant leadership (Di Fabio and Peiró, 2018). Sustainability leadership aspires to create sustainable learning conditions. It develops rather than exhausts human resources, supports workers in their development, and identifies resources, excluding the superfluous in critical aspects of work. Ethical leadership aims to promote fair goals, align actions with ideals, and enforce ethical standards. It empowers organization members, encouraging kindness, compassion, and care for others. Mindful leadership regards comprehension of collaborators, anticipating their requests, and being aware of their limitations and strengths. It recognizes the importance of managing personal emotions, especially in stressful situations. Finally, servant leadership recognizes the moral responsibility of the leader toward collaborators. It supports and assists them in identifying their needs and interests. In this perspective, all the leadership styles enclosed in HCSL involve the awareness of oneself and others, balancing different intrapersonal, interpersonal, and organizational aspects. These aspects of intrapersonal, interpersonal, and organizational awareness are based on an in-depth understanding of oneself and integrating the self in relationships. It is therefore promising to examine the potential of the relationships between PRM and such a current construct as HCSL. Furthermore, it has been established that personality traits, such as those contained in the Big Five personality model, have a significant relationship with a wide variety of human behaviors (Keefer et al., 2018), also showing a relationship with an array of leadership styles (e.g., Judge et al., 2002; Özbağ, 2016; Parr et al., 2016). Thus, it could be relevant to control for the effects of personality traits and verify whether PRM also explains additional variance beyond personality traits in relation to HCSL. It adds to the importance of PRM since it is conceived to be increased *via* specific training (Di Fabio, 2016; Di Fabio and Kenny, 2016a; Di Fabio and Kenny, 2016b), differently from personality traits considered essentially stable (Ferguson, 2010).

According to the delineated framework, the present study aimed at analyzing the associations between PRM and HCSL, taking into account personality traits. Specifically, the following hypotheses were formulated:

*H1*: A PRM and HCSL will be positively associated.

*H2*: In the relationship between PRM and HCSL, PRM will increase the percentage of explained variance in addition to the variance explained by personality traits.

## Materials and methods

### Participants

One hundred and ninety-one workers from Central Italy (49.21% males and 50.79% females; mean age = 44.97 years, *SD* = 12.71) participated in the study.

### Measures

Big five questionnaire (BFQ; Caprara et al., 1993): a self-report questionnaire composed of 132 items ranging from 1 = “*Absolutely false*” to 5 = “*Absolutely true*”. It measures five personality traits: extraversion (example of item: “It’s easy for me to talk to people I do not know”); agreeableness (example of item: “I almost always know how to meet the needs of others”); conscientiousness (example of item: “Before submitting an assignment, I spend a lot of time reviewing it”); emotional stability (example of item: “Usually it does not happen to me to react exaggerated even to strong emotions”); and openness (example of item: “Every novelty fascinates me”) (Caprara et al., 1993). Cronbach’s alphas reliability coefficients were from 0.75 (Openness) to 0.90 (Emotional stability).

PRM Scale (PRMS; Di Fabio, 2016): 12-item self-report scale ranked between 1 “*Strongly disagree*” and 5 = “*Strongly agree*”; Cronbach’s alphas reliability coefficients ranged from 0.80 (Caring) to 0.81 (Connectedness). PRMS comprises three factors: respect, caring and connectedness. Example of item are for the respect (e.g., “I have respect for the value and uniqueness of others”), caring (e.g., “Others often take care of me”), and connectedness (e.g., “I keep a balance in my relationships between family, friends and significant others”; Di Fabio, 2016).

HCSL Scale (HCSLS; Di Fabio and Peiró, 2018): 16-item self-report questionnaire with response format ordered from 1 = “*None*” to 5 = “*Very much*”. Examples of items are: “I act by giving an example of doing tasks in an ethically correct manner” (ethical leadership); “I create sustainable learning conditions that I take care to preserve” (sustainable leadership); “I put myself in the shoes of my co-workers when they are doing tasks” (mindful leadership); and “I encourage my collaborators when I realize that they encounter difficulties” (servant leadership; Di Fabio and Peiró, 2018). Cronbach’s alpha 0.94.

### Procedure

The administration of the self-rating scales was conducted collectively by specialized personnel, asking for written and informed consent according to privacy Italian laws (DL-196/2003; EU 2016/679). The sequence of the instruments’ administration was counterweighted to account the presentation order effects.

### Data analysis

Descriptive statistics, Pearson’s *r* correlations, and the hierarchical regression were conducted through SPSS software.

## Results

Table 1 shows Pearson’s *r* correlations among BFQ, PRMS and HCSLS.

**Table 1:** Correlations among big five questionnaire, positive relational management scale and human capital sustainability leadership scale.

	*M*	*SD*	1	2	3	4	5	6	7	8	9
1. Extraversion	74.92	10.24	-								
2. Agreeableness	78.15	10.28	0.12	-							
3. Conscientiousness	83.14	10.77	0.45**	0.20**	-						
4. Emotional stability	69.42	14.07	0.11	0.14	0.26**	-					
5. Openness	81.19	9.87	0.40**	0.48**	0.46**	0.27**	-				
6. Respect	16.19	2.53	0.27**	0.20**	0.25**	0.24**	0.22**	-			
7. Caring	14.53	2.58	0.20**	0.10	0.15*	0.16*	0.07	0.51**	-		
8. Connectedness	17.10	2.80	0.13	0.27**	0.18*	0.10	0.24**	0.50**	0.48**	-	
9. HCSLS	65.93	8.89	0.32**	0.26**	0.27**	0.14*	0.30**	0.44**	0.31**	0.45**	-

*N* = 191. * < 0.05, ** < 0.01. HCSLS, human capital sustainability leadership scale.

In Table 2 the findings of hierarchical regression conducted using the HCSL as dependent variable are reported. At step 1, Personality traits explained the 17% of the variance; at step 2, the PRMS dimensions increased 16% of the variance. Overall, the model explained the 33% of the variance.

**Table 2:** Hierarchical regression: contribution of personality traits (big five questionnaire) and positive relational management scale in relation to human capital sustainability leadership scale.

	HCSLS β
**Step 1**	
Extraversion	0.22**
Agreeableness	0.18*
Conscientiousness	0.10
Emotional stability	0.05
Openness	0.06
**Step 2**	
Extraversion	0.17*
Agreeableness	0.09
Conscientiousness	0.06
Emotional stability	0.01
Extraversion	0.04
Respect	0.21*
Care	0.01
Connectedness	0.28**
*R*^2^ step 1	0.17***
Δ*R*^2^ step 2	0.16***
*R*^2^ total	0.33***

*N* = 191; * < 0.05, ** < 0.01. ***p < 0.001. HCSLS, human capital sustainability leadership scale.

## Discussion

The current research sought to analyze the relationships between PRM and HCSL, taking into account personality traits. A statistically significant and positive association was observed between PRM and HCSL (*H1*), also after controlling for personality traits (*H2*), confirming the hypotheses of this research. The dimensions of PRM that particularly emerged related to HCSL are Connectedness followed by Respect. The findings highlighted that the aspects of PRM of workers relative to connectedness and reciprocity with others and respect (for others, of others for me, for myself; Di Fabio, 2016) were related to a leadership style centered on healthy people as resilient and flourishing workers (Di Fabio and Peiró, 2018). These results emphasize positive relationships characterized by connectedness and the positive constellation of respect towards others, of others towards me and my respect towards myself at the workplace are associated with a higher HCSL style. Overall, these results underlined the value of PRM in relation to HCSL in organizations (Di Fabio and Peiró, 2018). Furthermore, since Connectedness and Respect emerged as PRM dimensions mainly associated with HCSL, these two dimensions could be assessed with attention in research and intervention aimed at studying and fostering PRM to promote HCSL. It could also be relevant for the accountability framework, which encourages researchers to use evidence-based methodologies to ensure a balance in cost-effectiveness (Whiston, 1996, 2001). Tailored strategy focused on specific dimensions could be a promising strategy for decreasing the time and costs of research and intervention (Whiston, 1996, 2001).

## Limits and conclusion

This study has some limits. Participants were workers of Central Italy not representative of all Italian workers. In future research, workers of different Italian regions should be involved to examine the relationships between the constructs included in this study. Furthermore, it could be worthy to replicate this research in international contexts also cross-culturally. A further limit is relative to the cross-sectional design of the research that calls for future longitudinal research. Future studies could expand the knowledge concerning the associations between PRM and HCSL, for example, investigating the moderating or the mediating role of PRM on the relationship between personality traits and HCSL, with a particular focus on the Respect and Connectedness dimensions.

Even though these limits and the necessity of additional research, these results expand the literature highlighting the role of PRM in contributing to HCSL. If the findings of this study are confirmed, new opportunities for interventions will emerge regarding HCSL. Preventive perspectives underline the relevance of managing positive relationships (Di Fabio, 2016) to enhance both relationality (Blustein, 2011) and respectivity (Maree, 2012) for flourishing of people in work and life contexts (Di Fabio, 2014). Furthermore, in strengths-based prevention (Di Fabio and Saklofske, 2021) as well as in primary prevention perspectives (Hage et al., 2007), PRM could have a preventive value in relation to a leadership style that promote human resources sustainability and sustainable development (Di Fabio and Peiró, 2018).

Both PRM and HCSL constitute precious resources for building well-being at the workplace (Cartwright and Cooper, 2014; Johnson et al., 2018), and crucial assets to enable flourishing of healthy workers, healthy business, and healthy organizations (Di Fabio et al., 2020).

## Data availability statement

The raw data supporting the conclusions of this article will be made available by the authors, without undue reservation.

## Ethics statement

The studies involving human participants were reviewed and approved by the Ethics Committee of the Integrated Psychodynamic Psychotherapy Institute (IPPI). The patients/participants provided their written informed consent to participate in this study.

## Author contributions

ADF conceptualized the paper, supervised and tutored AB. AB realized the investigation and run statistical analyses. LP and AS wrote the first draft of the paper. ADF, AG, and AS reviewed, edited, and wrote the final draft of the paper. All authors contributed to the article and approved the submitted version.

## Conflict of interest

The authors declare that the research was conducted in the absence of any commercial or financial relationships that could be construed as a potential conflict of interest.

## Publisher’s note

All claims expressed in this article are solely those of the authors and do not necessarily represent those of their affiliated organizations, or those of the publisher, the editors and the reviewers. Any product that may be evaluated in this article, or claim that may be made by its manufacturer, is not guaranteed or endorsed by the publisher.
